# Region- and Cell-Specific Expression of Transmembrane Collagens in Mouse Brain

**DOI:** 10.3389/fnint.2017.00020

**Published:** 2017-08-30

**Authors:** Aboozar Monavarfeshani, Courtney N. Knill, Ubadah Sabbagh, Jianmin Su, Michael A. Fox

**Affiliations:** ^1^Developmental and Translational Neurobiology Center, Virginia Tech Carilion Research Institute Roanoke, VA, United States; ^2^Department of Biological Sciences, Virginia Tech Blacksburg, VA, United States; ^3^Virginia Tech Carilion School of Medicine, Virginia Tech Roanoke, VA, United States; ^4^Translational Biology, Medicine, and Health Graduate Program, Virginia Tech Blacksburg, VA, United States; ^5^Department of Pediatrics, Virginia Tech Carilion School of Medicine Roanoke, VA, United States

**Keywords:** extracellular matrix, collagen, retina, superior colliculus, lateral geniculate nucleus, accessory olfactory bulb, hippocampus, interneuron

## Abstract

Unconventional collagens are nonfribrillar proteins that not only contribute to the structure of extracellular matrices but exhibit unique bio-activities. Although roles for unconventional collagens have been well-established in the development and function of non-neural tissues, only recently have studies identified roles for these proteins in brain development, and more specifically, in the formation and refinement of synaptic connections between neurons. Still, our understanding of the full cohort of unconventional collagens that are generated in the mammalian brain remains unclear. Here, we sought to address this gap by assessing the expression of transmembrane collagens (i.e., collagens XIII, XVII, XXIII and XXV) in mouse brain. Using quantitative PCR and *in situ* hybridization (ISH), we demonstrate both region- and cell-specific expression of these unique collagens in the developing brain. For the two most highly expressed transmembrane collagens (i.e., collagen XXIII and XXV), we demonstrate that they are expressed by select subsets of neurons in different parts of the brain. For example, collagen XXIII is selectively expressed by excitatory neurons in the mitral/tufted cell layer of the accessory olfactory bulb (AOB) and by cells in the inner nuclear layer (INL) of the retina. On the other hand, collagen XXV, which is more broadly expressed, is generated by subsets of excitatory neurons in the dorsal thalamus and midbrain and by inhibitory neurons in the retina, ventral thalamus and telencephalon. Not only is *col25a1* expression present in retina, it appears specifically enriched in retino-recipient nuclei within the brain (including the suprachiasmatic nucleus (SCN), lateral geniculate complex, olivary pretectal nucleus (OPN) and superior colliculus). Taken together, the distinct region- and cell-specific expression patterns of transmembrane collagens suggest that this family of unconventional collagens may play unique, yet-to-be identified roles in brain development and function.

## Introduction

Collagens are triple helical extracellular matrix (ECM) proteins assembled from three separate polypeptide (α) chains, each of which contains one or more repeating G-X-Y peptide sequences (termed collagenous domains). Over 70 genes in mammals encode collagen α chains or collagen-like molecules, which are related molecules that contain collagenous domains but were named for other protein domains or functions (such as gliomedin, complement C1q, and the collagenous tail of acetylcholinesterase (ColQ; Fox, 2008; Ricard-Blum, 2011). Structural roles of collagen molecules, especially those assembled into fibrillar supramolecular complexes, have been well-studied, however, many collagens and collagen-like molecules are not assembled into such structural fibrils and instead exhibit unique bio-activities and biological functions (Ricard-Blum, 2011; Mouw et al., 2014). These “unconventional” collagens (which refer to as non-fibrillar collagens) contain thrombospondin-like domains, EMI-like domains (i.e., cysteine rich domains found in emilin and multimerin), fibronectin repeats, von Willebrand factor A-like domains, and matricryptin domains (i.e., domains that are proteolytically shed from full-length matrix proteins and exhibit unique bioactivities once shed; Ricard-Blum, 2011; Ricard-Blum and Ballut, 2011). Moreover, a subset of collagens, including collagens XIII, XVII, XXIII and XXV, are type II membrane proteins with large ecto-domains and are aptly termed membrane associated collagens with interrupted triple helices (MACITs; Pihlajaniemi and Rehn, 1995; Hägg et al., 1998; Hashimoto et al., 2002; Banyard et al., 2003). MACITs exist in either full-length transmembrane forms or soluble, extracellular forms that are generated through ecto-domain shedding by extracellular proteases such as furin, matrix metalloproteinases, and ADAMs (a disintegrin and metalloproteinases; Franzke et al., 2002, 2004; Hashimoto et al., 2002; Vaisanen et al., 2004; Veit et al., 2007).

Bio-active roles of unconventional collagens in the formation of neural circuits have been identified in the peripheral nervous system (Fox, 2008). At the neuromuscular junction (NMJ), a peripheral synapse formed between skeletal muscles and spinal motor neurons, a dense basal lamina lies within the cleft separating pre- and postsynaptic elements. A number of unconventional collagens, including MACITs, are present in this synaptic basal lamina and are necessary for the development and function of the NMJ (Miner and Sanes, 1994; Feng et al., 1999; Fox et al., 2007; Latvanlehto et al., 2010; Su et al., 2012). For example, muscle-derived collagen XIII is enriched in the synaptic basal lamina and mutations in *col13a1* (the gene encoding collagen XIII) are associated with congenital myasthenic syndrome (CMS) type 19 (Logan et al., 2015). Loss of collagen XIII in mice not only phenocopies CMS type 19, but leads to impaired maturation of the postsynaptic apparatus (Latvanlehto et al., 2010; Härönen et al., 2017). Presynaptic defects and withdrawal of motor nerve terminals from synaptic sites are present in collagen XIII-deficient mice, but the development of these phenotypes is prevented in mice that lack only the ecto-domain shed form of collagen XIII, suggesting that transmembrane collagen XIII may contribute to synaptic adhesion (Härönen et al., 2017). Furthermore, another MACIT, collagen XXV, is generated by both spinal motor neurons and developing skeletal muscle fibers and its loss impairs intramuscular growth of motor axons and leads to a failure of motor neuron survival (Tanaka et al., 2014). Recessive mutations in human *col25a1* (the gene encoding collagen XXV) are associated with a novel congenital cranial dysinnervation disorder (CCDD), a condition affecting the innervation and function of extraocular muscles (Shinwari et al., 2015).

In the mammalian brain, several matricyptin-releasing collagens and collagen-like molecules similarly contribute to neural circuit formation (Stevens et al., 2007; Su et al., 2010, 2012, 2016; Bialas and Stevens, 2013). Roles for MACITs in the brain, however, remain less clear, despite reports suggesting they are present in the mammalian brain (Hashimoto et al., 2002; Claudepierre et al., 2005; Koch et al., 2006; Seppänen et al., 2006, 2007; Dennis et al., 2010). Therefore, in the present study, we sought to provide a detailed description of the region- and cell-specific expression patterns of *col13a1, col17a1, col23a1* and *col25a1* (the genes encoding all four MACIT collagens) in the developing mouse brain. Our results demonstrate for the first time that transmembrane collagens are expressed in discrete population of neurons throughout the brain. For example, collagen XXIII is selectively expressed by a small subset of excitatory neurons in the accessory olfactory bulb (AOB). On the other hand, collagen XXV, which is more broadly expressed throughout the brain, is generated by inhibitory neurons in the ventral thalamus, midbrain and telencephalon and by excitatory neurons in dorsal thalamus. Taken together, the distinct, region- and cell-specific patterns of MACIT expression in the brain suggests that this family of unconventional collagens may play unique, yet-to-be identified roles in brain development and function.

## Experimental Procedures

### Animals

CD1 and C57BL/6 mice were obtained from Charles River Laboratories (Wilmington, MA, USA). *Parv-cre*, *thy1-stop-yfp* (line15), *rosa-stop-tdt* (Ai9) and *calb2-cre* were obtained from Jackson Labs (stock numbers 008069, 005630, 007905 and 010774, respectively). *Aldh1l1-gfp* mice were generated from GENSAT project and were obtained from Dr. Stefanie Robel (VTCRI; also available from MMRRC). Genomic DNA was isolated from tails using the HotSHOT method (Truett et al., 2000), and genotyping was performed with the following primers: *gfp* (yfp), 5′-AAG TTC ATC TGC ACC ACC G-3′ and 5′-TCC TTG AAG AAG ATG GTG CG-3′; *cre*, 5′-TGC ATG ATC TCC GGT ATT GA-3′ and 5′-CGT ACT GAC GGT GGG AGA AT-3′; *tdt*, 5′-ACC TGG TGG AGT TCA AGA CCA TCT-3′ and 5′-TTG ATG ACG GCC ATG TTG TTG TCC-3′. Primers were purchased from Integrated DNA Technologies (Coralville, IA). The following cycling conditions were used for yfp: 35 cycles using a denaturation temperature of 94°C for 30 s, annealing at 58°C for 1 min, and elongation at 72°C for 45 s. The following cycling conditions were used for cre: 35 cycles using a denaturation temperature of 95°C for 30 s, annealing at 60°C for 30 s, and elongation at 72°C for 45 s. All analyses conformed to National Institute of Health guidelines and protocols approved by the Virginia Polytechnic Institute and State University Institutional Animal Care and Use Committee.

### Reagents

All chemicals and reagents were purchased from Fisher (Fairlawn, NJ, USA) or Sigma (St. Louis, MO, USA) unless otherwise stated.

### Antibodies

Antibodies used in these studies were as follows: rabbit anti-calbindin (diluted 1:2500 for immunohistochemistry (IHC); Swant), rabbit anti-calretinin (diluted 1:1000 for IHC; Millipore), rabbit anti-GFP (diluted 1:500; Life Technologies), mouse anti-GAD67 (diluted 1:1000 for IHC; Millipore), rabbit anti-Iba1 (diluted 1:500 for IHC, WAKO), rabbit anti-Vasoactive Intestinal Peptide (VIP; diluted 1:150 for IHC; Immunostar). All fluorescent secondary antibodies for IHC were from Life Technologies (diluted 1:1000).

### Immunohistochemistry

Fluorescent IHC was performed on 16 μm cryosectioned 4% paraformaldehyde (PFA)-fixed brain tissue (*n* = 3 mice) as described previously (Fox et al., 2007; Su et al., 2012). Briefly, tissue slides were allowed to air dry for 15 min before being incubated with blocking buffer (2.5% normal goat serum, 2.5% bovine serum albumin and 0.1% Triton X-100 in PBS) for 1 h. Primary antibodies were diluted in blocking buffer and incubated on tissue sections overnight at 4°C. On the following day, tissue slides were washed in PBS and secondary antibodies (diluted 1:1000 in blocking buffer) were applied to slides for 1 h at room temperature. After thoroughly washing in PBS, tissue slides were coverslipped with VectaShield (Vector Laboratories, Burlingame, CA, USA). Images were acquired on a Zeiss LSM 700 confocal microscope (Oberkochen, Germany).

### *In Situ* Hybridization

*In situ* hybridization (ISH) was performed on 16 μm coronal or sagittal cryosectioned tissues (*n* = 3 mice) as previously described (Fox and Sanes, 2007; Su et al., 2010). The antisense riboprobes were generated from full-length *col25a1* and *syt1* Image clones (MMM1013-202707906 [col25a1]; MM1013-9199901 [syt1] from Dharmacon). Riboprobes were also generated against 1 kb fragments of *col23a1* (corresponding to nucleotides 839–1934) and *gad1* (corresponding to nucleotides 1099–2081) which were polymerase chain reaction (PCR)-cloned into pGEM Easy T vector (Promega, Madison, WI) with the following primers: *col23a1*, 5′-AGA TGG AGT TGC AGG ACC AC-3′ and 5′-TCA CTT ATG CCA GCA ACC AG-3′; *gad1*, 5′-TGT GCC CAA ACT GGT CCT -3′ and TGG CCG ATG ATT CTG GTT-3′. Briefly, riboprobes were synthesized using digoxigenin (DIG)- or fluorescein (FL)-labeled UTP (Roche, Mannheim, Germany) and the MAXIscript *in vitro* Transcription Kit (Ambion, Austin, TX, USA). Probes were hydrolyzed to 500 nt. Coronal or sagittal brain and retina sections were hybridized with riboprobes at 65°C as previously described (Su et al., 2010). Bound riboprobes were detected by horseradish peroxidase (POD)-conjugated anti-DIG or anti-FL antibodies followed by fluorescent staining with Tyramide Signal Amplification (TSA) systems (PerkinElmer, Shelton, CT, USA). Images were obtained on a Zeiss Axio Imager A2 fluorescent microscope or a Zeiss Examiner Z1 LSM 700 confocal microscope. A minimum of three animals per genotype and age was compared in ISH experiments.

### Quantitative Real-Time PCR

RNA from cerebral cortex (Ctx; P21), dorsal lateral geniculate nucleus (dLGN; of the dorsal thalamus; P21), hippocampus (P0, P7, P15, P21, P60), superior colliculus (P21), olfactory bulb (P21) and retina (P21) (*n* = 3, each pooled from 3 to 5 mice) was isolated using the Fibrous and Fatty Tissue RNA extraction kit (BioRad). cDNAs were generated from 250 ng RNA with Superscript II Reverse Transcription First Strand cDNA Synthesis kit (Invitrogen). Quantitative real-time PCR (qPCR) was performed on a CFX Connect Real-Time system (BioRad) using iTag SYBRGreen Supermix (BioRad) by the following primers: *col25a1*, 5′- GAT TCT CCT CTT CGG CCT CT -3′and 5′- AAA TAA GAA CGG CCA GGG AG -3′; *col23a1*, 5′- GCA ATC AGG ACG AGA TGG CT-3′ and 5′- AAA GTC TCC CGG TGT ACC CT-3′; *col17a1*, 5′-TGG GAT CAG CTT TGG GCA TC-3′ and 5′- GAC AAA CCA GCG GCT CGG A-3′; *col13a1*, 5′- AAG GGA GAA GCA GGC CTA GAG-3′ and 5′-TGG AGT ACC AGG CAA TCC CAG-3′; *gapdh*, 5′-CGT CCC GTA GAC AAA ATG GT-3′ and 5′-TTG ATG GCA ACA ATC TCC AC-3′. The following cycling conditions were used with 12.5 ng of cDNA: 95°C for 30 s, followed by 40 cycles of amplification (95°C for 5 s, 60°C for 30 s, 55°C for 60 s) and a melting curve analysis. Relative quantities of RNA were determined using the 2^−ΔCT^ method (Livak and Schmittgen, 2001).

### RNAseq

RNA from dLGN and ventral lateral geniculate nucleus (vLGN) at four different developmental time points (P3, P8, P12 and P25) were isolated using the Fibrous and Fatty Tissue RNA extraction kit (*n* = 4–5, each pooled from 5 to 7 mice) and send to the Genomics Research Laboratory at Virginia Tech’s Biocomplexity Institute for RNAseq analysis. Stranded RNAseq library construction was performed on Apollo 324 Robot (Wafergen, Fremont, CA, USA). Five hundred nanogram total RNA with RIN ≥8.0 was enriched for polyA RNA using PrepX PolyA mRNA Isolation Kit (P/N 400047, Wafergen, Fremont, CA, USA) and converted into a library of template molecules using PrepX RNA-Seq for Illumina Library Kit (P/N 400046, Wafergen, Fremont, CA, USA). The 280–300 bp libraries (160–180 bp insert) were validated using Agilent 2100 Bioanalyzer and were quantitated using Quant-iT dsDNA HS Kit (Invitrogen) and qPCR. Eight individually indexed cDNA libraries were pooled and sequenced on Illumina HiSeq and a minimum of 40–50 million reads were obtained. Finally, libraries were clustered onto a flow cell using Illumina’s TruSeq PE Cluster Kit v3-cBOT-HS (PE-401-3001), and sequenced 2 × 100 PE using TruSeq SBS Kit v3-HS for 200-cycles (FC-401- 3001). DESeq2 was used for all data analysis.

## Results

### Region-Specific Expression of MACIT Collagens in Mouse Brain

**Figure 1:**
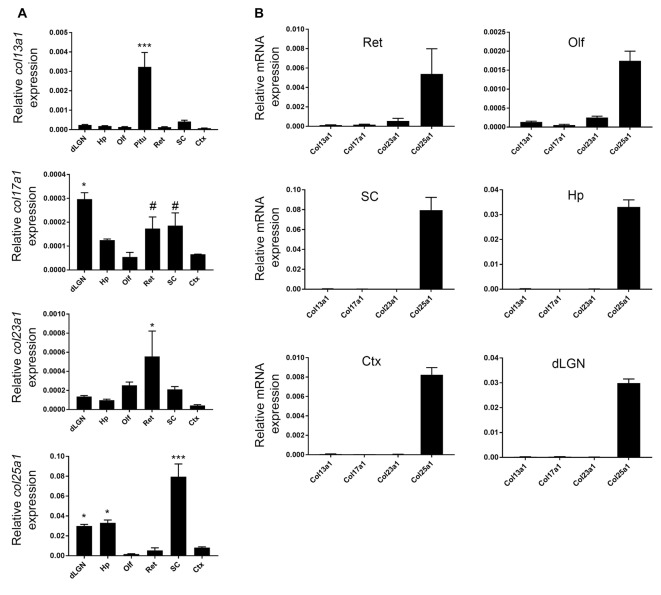
Region-specific membrane associated collagens with interrupted triple helices (MACIT) mRNA expression in mouse brain. **(A)** Quantitative RT-PCR (QPCR) analysis of *col13a1, col17a1, col23a1* and *col25a1* mRNA levels in different regions of postnatal day 21 (P21) mouse brain and retina. Levels of MACIT mRNA expression are normalized to *gapdh* in each sample. Data shown represent means +/− standard error of the mean (SEM; error bars). ***Indicates MACIT mRNA expression in that region differs from all other regions by *p* < 0.001 by ANOVA with Uncorrected Fisher’s LSD test. For *col17a1* mRNA, *Indicate differs from all other regions by *p* < 0.05 and ^#^Indicate differs from expression in Ctx, Olf and dorsal lateral geniculate nucleus (dLGN) by *p* < 0.05 both by ANOVA with Uncorrected Fisher’s LSD test. For *col23a1* mRNA, *Indicate differs from expression in Ctx, dLGN, Hp and SC by *p* < 0.05 by ANOVA with Uncorrected Fisher’s LSD test. For *col25a1* mRNA, *Indicate differs from expression in Ctx, Olf and retina by *p* < 0.05 by ANOVA with Uncorrected Fisher’s LSD test. *n* = 3 samples per region. **(B)** Comparison of different MACIT mRNAs in each brain region. Levels of MACIT mRNA expression are normalized to *gapdh* in each sample. Ctx, cerebral cortex; dLGN, dorsal lateral geniculate nucleus; Hp, hippocampus; Olf, olfactory bulb; Pit, pituitary gland; SC, superior colliculus.

To assess region-specific expression patterns of *col13a1, col17a1, col23a1* and *col25a1*, at times correlating with neural circuit development and maturation, we performed qPCR on RNA isolated from several regions of P21 mouse brain. These regions included the Ctx, dLGN (of the dorsal thalamus), hippocampus, superior colliculus, olfactory bulb, and retina. Each MACIT exhibited a unique pattern of mRNA expression in these regions: the highest level of *col25a1* was observed in superior colliculus, while the highest levels of *col17a1* and *col23a1* were observed in thalamus and the retina, respectively (Figure 1A). Levels of *col13a1* mRNA were low in all brain regions examined, especially when compared to other tissues, such as the pituitary gland (Figure 1A). It is important to note, when compared against each other, *col25a1* mRNA appeared to be expressed at dramatically higher levels than other MACITs in all brain regions analyzed (Figure 1B). This result, that *col25a1* mRNA is expressed at dramatically higher levels than other MACITs, matches unbiased transcriptional profiling performed in both thalamus and superior colliculus (data not shown).

### *Col23a1* mRNA Is Generated by Neurons in the Retina and Accessory Olfactory Bulb

**Figure 2:**
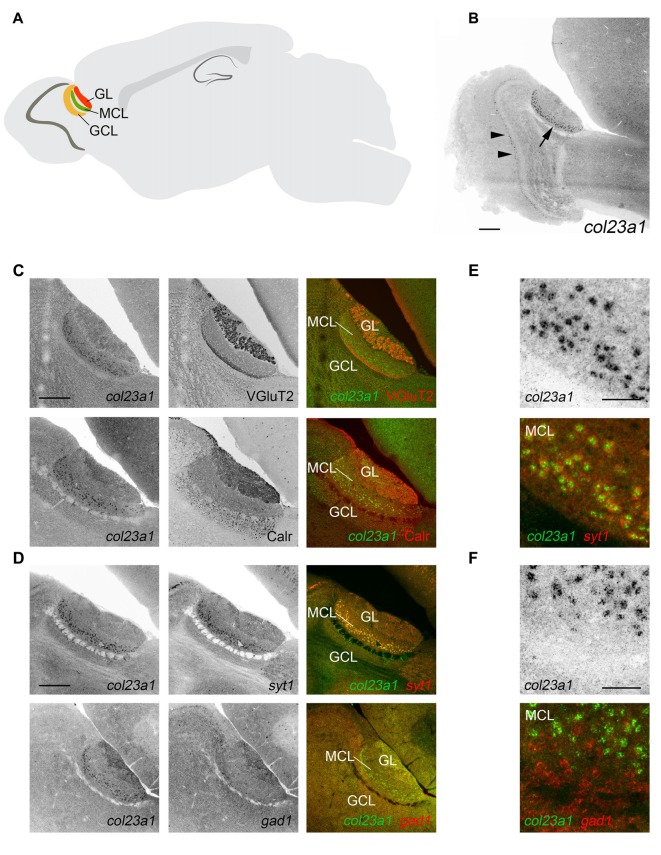
Excitatory neurons in the mitral cell layer of the accessory olfactory bulb (AOB) generate col23a1 mRNA. **(A)** Schematic depicting the location and layers of the AOB. GL, glomerular layer; MCL, mitral cell layer; GCL, granule cell layer. **(B)** *In situ* hybridization (ISH) for *col23a1* mRNA revealed layer-specific expression in the AOB (arrow). Sporadic expression was also observed in the MCL of the main olfactory bulb (MOB; arrow heads). **(C)** ISH coupled to immunostaining for VGluT2 or Calr, revealed expression of *col23a1* in the MCL of the AOB. **(D–F)** Double ISH with riboprobes against *col23a1* and either *syt1* or *gad1*, revealed excitatory, and not inhibitory, neurons generate this MACIT. **(E,F)** depict high magnification images of *col23a1* expression in *syt1*-expressing neurons, but not in *gad1*-expressing interneurons. Scale bar in **(B)** = 400 μm, in **(C)** = 200 μm, in **(D)** = 200 μm and in **(E)** and **(F)** = 50 μm.

**Figure 3:**
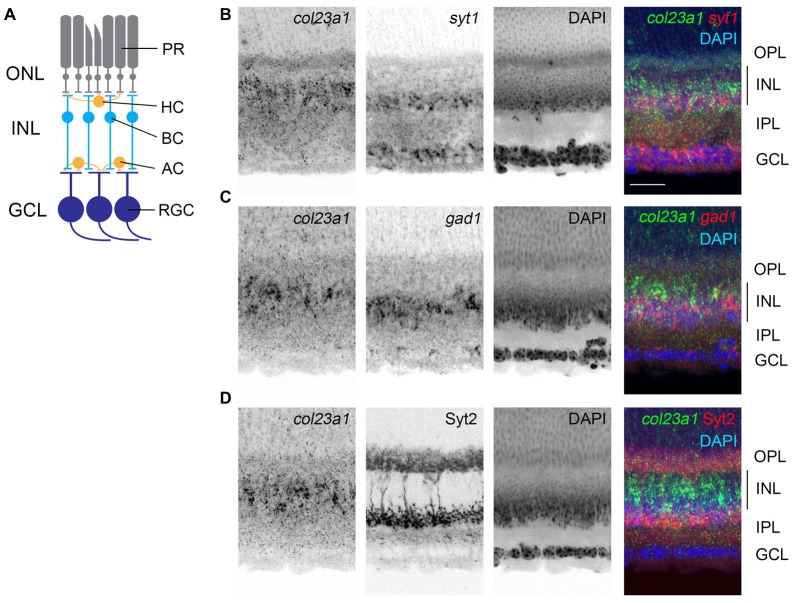
Cells in the central inner plexiform layer of the retina express *col23a1* mRNA. **(A)** Schematic representation of the mammalian retina. PR, photoreceptors; ONL, outer nuclear layer; OPL, outer plexiform layer; INL, inner nuclear layer; HC, horizontal cell; BC, bipolar cell; AC, amacrine cell; IPL, inner plexiform layer; RGC, retinal ganglion cell; GCL, ganglion cell layer. **(B,C)** Double ISH with riboprobes against *col23a1* and either *syt1* or *gad1*. Expression of *col23a1* in cells that were largely distributed above the *syt1*- and *gad1*-expressing amacrine cells in the inner most sublayer of the IPL. **(D)** ISH shows that *col23a1* mRNA is expressed in cells in the central region of the IPL, below Syt2-immunoreactive OFF-bipolar cells. Scale bar = 50 μm.

To more precisely understand the cell-specific expression of MACITs in mouse brain, we focused our attention on *col23a1* and *col25a1*, two MACITs which appear highly expressed in brain but that we know little, if anything, about their cellular origin. To determine the cell-specific expression of *col23a1* mRNA, we generated riboprobes against a ~1 kB fragment of *col23a1* and performed ISH on sagittal sections of mouse brain. In contrast to the qPCR results, ISH revealed significant cellular expression of *col23a1* mRNA in just two brain regions—the retina and the AOB, a component of the vomeronasal system that contributes to the processing of pheromone signals (Figures 2, 3).

Located within the superior-caudal region of the olfactory bulb, the AOB is a multi-layered structure that consists of an outer glomerular layer (GL), an external plexiform layer (EPL), a mitral/tufted cell layer (MCL), an internal plexiform layer (IPL) and a granule cell layer (GCL; Figure 2A). To determine which layer of AOB contained *col23a1-*expressing cells, we combined ISH for *col23a1* mRNA with immunostaining for vesicular glutamate transporter 2 (VGluT2), which labels synaptic terminals in the GL and IPL, and calretinin, which labels periglomerular cells in the GL and granule cells (Jia and Halpern, 2004; Yokosuka, 2012). *Col23a1*-expressing cells were located between regions of significant VGluT2- and Calr- immunoreactivity, indicating they were within the MCL of the AOB (Figure 2C). Weak expression of *col23a1* mRNA was also observed sporadically in the MCL of the main olfactory bulb (MOB; Figure 2B).

The MCL of the AOB is a neuron-dense region. To determine if *col23a1-*expressing cells were neurons, we performed double ISH (D-ISH) for *col23a1* and *syt1*, a neuronally expressed gene that encodes the calcium sensor synaptotagmin 1 (Syt1), an integral component of the presynaptic machinery needed for neurotransmitter release. *Col23a1*-expressing cells in the adult MCL of the AOB co-expressed *syt1*, indicating they were neurons (Figure 2D). As a set of inhibitory interneurons reside with the MCL and EPL of the olfactory bulb (Huang et al., 2013), we next tested whether *col23a1* mRNA was generated by inhibitory neurons. To accomplish this, we generated riboprobes directed against *gad1*, the gene that encodes the enzyme glutamate decarboxylase 67 (GAD67). We failed to observe any *col23a1*-expressing cells that also generated *gad1* mRNA (Figure 2D). Thus, *col23a1* mRNA appears selectively expressed by excitatory mitral or tufted neurons in the MCL of the mouse AOB.

Like the AOB, the retina is also a multi-layered neural structure. Lying within the posterior chamber of the eye, the retina’s main function is to transduce light-derived information into neural signals (Figure 3A). Photoreceptors, in the outer most portion of the retina, transduce light-derived stimuli into neural signals and transmit these signals to a series of retinal interneurons in the inner nuclear layer (INL) of the retina (i.e., bipolar cells, amacrine cells, and horizontal cells). Bipolar cells relay these light-derived signals to retinal ganglion cells (RGCs), which project axons through the optic nerve to retino-recipient regions of the brain. ISH with riboprobes against *col23a1* revealed significant expression of this MACIT collagen only in the INL of the retina (Figure 3B). Interneurons are regionally localized in the INL, with horizontal and bipolar cells residing adjacent to the outer plexiform layer (OPL) and amacrine cells residing adjacent to the inner plexiform layer (IPL; Figure 3A). ISH revealed that *col23a1* mRNA was distributed in the central-most region of INL, in a region between *syt1+* and *gad1+* amacrine cells and synaptotagmin 2 (Syt2)-expressing OFF-bipolar cells (Fox and Sanes, 2007; Figure 3C). While additional studies are needed to determine the exact cellular origin of *col23a1* in the retina, these results suggest it may be generated by ON-bipolar cells, a population of neurons that lie between inhibitory amacrine cells and OFF-bipolar cells (Figure 3C; Park et al., 2017).

### *Col25a1* mRNA Is Generated by Neurons in Regions of the Brain Associated with Visual Processing

**Figure 4:**
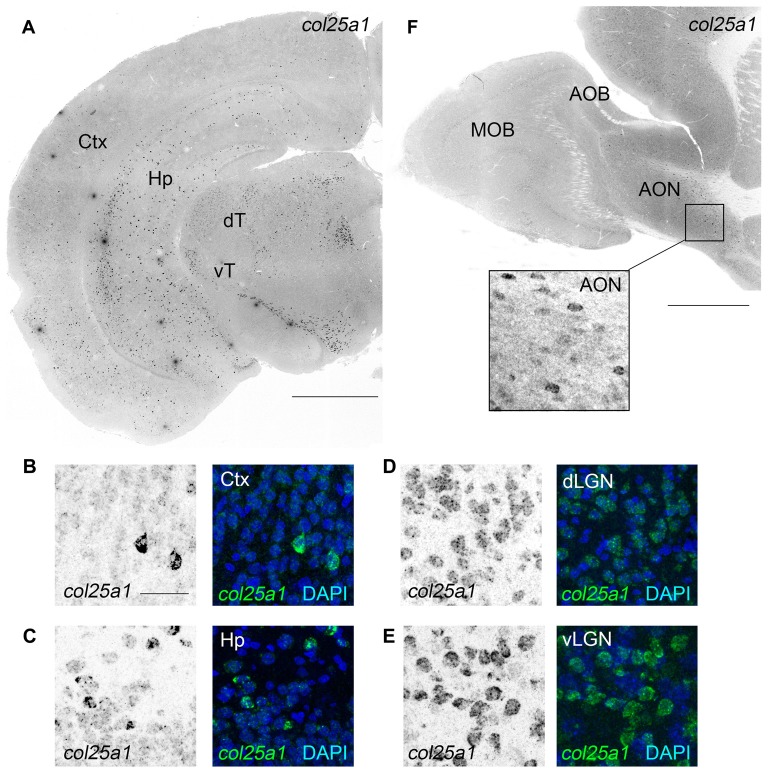
Widespread expression of *col25a1* mRNA in mouse brain. **(A)** Localization of *col25a1* mRNA by ISH in a coronal section of P25 mouse brain. Ctx, cerebral cortex; Hp, hippocampus; dT, dorsal thalamus; vT, ventral thalamus. **(B–E)** High magnification images of *col25a1*-expressing cells (labeled by ISH) in Ctx, Hp, dorsal lateral geniculate nucleus (dLGN) and ventral lateral geniculate nucleus (vLGN). Nuclei labeled with DAPI. **(F)** Localization of *col25a1* mRNA by ISH in a sagittal section of P60 mouse olfactory bulb. *Col25a1*-expressing cells were absent from the MOB and AOB, but were present in the anterior olfactory nucleus (AON; **F**). Scale bar = 1 mm for **(A,F)**; scale bar = 25 μm for **(B–E)** and inset in **(F)**.

**Figure 5:**
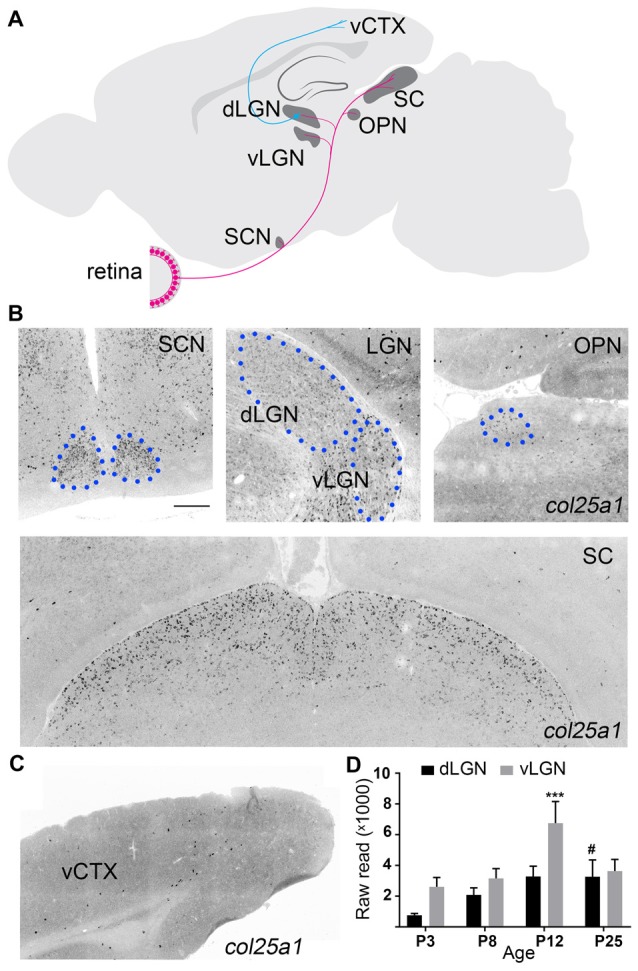
Brain regions associated with processing visual information display enriched *col25a1* expression. **(A)** Schematic depicting major retino-recipient nuclei that receive input from retinal ganglion cells (in pink). Relay cells in the dorsal lateral geniculate nucleus (dLGN) transmit visual information to primary visual cortex (vCTX). SCN, suprachiasmatic nucleus; vLGN, ventral lateral geniculate nucleus; OPN, olivary pretectal nucleus; SC, superior colliculus. **(B)** Localization of *col25a1* mRNA by ISH in retino-recipient nuclei of coronal sections of P25 mouse brain. SCN, dLGN, vLGN, and OPN are highlighted by blue circles. **(C)** Expression of *col25a1* mRNA in vCTX (of coronal sections of P25 mouse brain) is dramatically lower than in retino-recipient regions. **(D)** *col25a1* mRNA expression obtained by RNAseq in developing mouse dLGN and vLGN. ***Indicates *p* < 0.0001 comparing *col25a1* mRNA level in vLGN of P12 to vLGN of all other ages by ANOVA. ^#^Indicates *p* < 0.0001 comparing *col25a1* mRNA level in dLGN of P25 mice to P3 and P8 dLGN by ANOVA. *n* = 4–5 samples per age and region. Scale bar = 200 μm.

We next turned our attention to *col25a1*, and, as we did for *col23a1*, generated riboprobes against this MACIT. ISH revealed widespread *col25a1* mRNA expression throughout the telencephalon, diencephalon and brainstem (Figure 4), results that correlated well with qPCR data (Figure 1). For example, the highest level of *col25a1* mRNA expression in both qPCR and ISH was the superior colliculus, a large midbrain structure associate with visual processing and multisensory integration (Figures 4, 5). Likewise, by qPCR, expression of *col25a1* mRNA appeared lowest in the olfactory bulb and ISH revealed a lack of expression in both the MOB and the AOB (Figure 4B). Still, *col25a1* mRNA was present in the anterior olfactory nucleus (AON; also called the anterior olfactory cortex), a cortical region adjacent to the OB that is not only closely associated with the OB but was likely included in our OB RNA extracts (Figure 4B). It is noteworthy that the distribution of *col25a1* mRNA observed with our riboprobes is far less widespread than previously published work in which riboprobes were generated against a small fragment of the 3′UTR and initial coding sequence of *col25a1* (corresponding to nucleotides −267 to 333 of mouse col25a1; Hashimoto et al., 2002). However, not only does our ISH data correspond well with qPCR datasets (Figure 1) and RNAseq datasets (data not shown), it also closely resembles on-line ISH datasets that were generated with unique *col25a1* riboprobes (Lein et al., 2007)^1^. This gives us high confidence in the specificity of our *col25a1* riboprobes.

ISH analysis permitted us to take a global look at *col25a1* mRNA expression in mouse brain, and it revealed a striking feature of this MACITs distribution: enrichment of *col25a1* mRNA was observed in regions of brain associated with processing of visual information (Figure 5). As described above, the transduction of light-derived stimuli into neural signals occurs in the retina. Axons from the retina transmit these signals to over 40 different retino-recipient regions in the brain, most of which are scattered throughout the hypothalamus, thalamus, and midbrain (Morin and Studholme, 2014; Monavarfeshani et al., 2017; Figure 5A). Interestingly, expression of *col25a1* mRNA appeared highest in some of these retino-recipient regions, including the superior colliculus, vLGN, dLGN, intergeniculate leaflet (IGL), olivary pretectal nucleus (OPN) and suprachiasmatic nucleus (SCN; Figure 5B). Although expression of *col25a1* mRNA appeared highest in retino-recipient regions, it was not evenly expressed in each of these regions: expression appeared higher in the superior colliculus and SCN compared to the LGN and OPN (Figure 5B), results that closely matched qPCR data (Figure 1A). Moreover, the developmental expression of *col25a1* mRNA in these retinorecipient regions may also be region-specific. RNAseq analysis on dLGN and vLGN revealed unique developmental patterns of expression in each retinorecipient region (Figure 5D).

**Figure 6:**
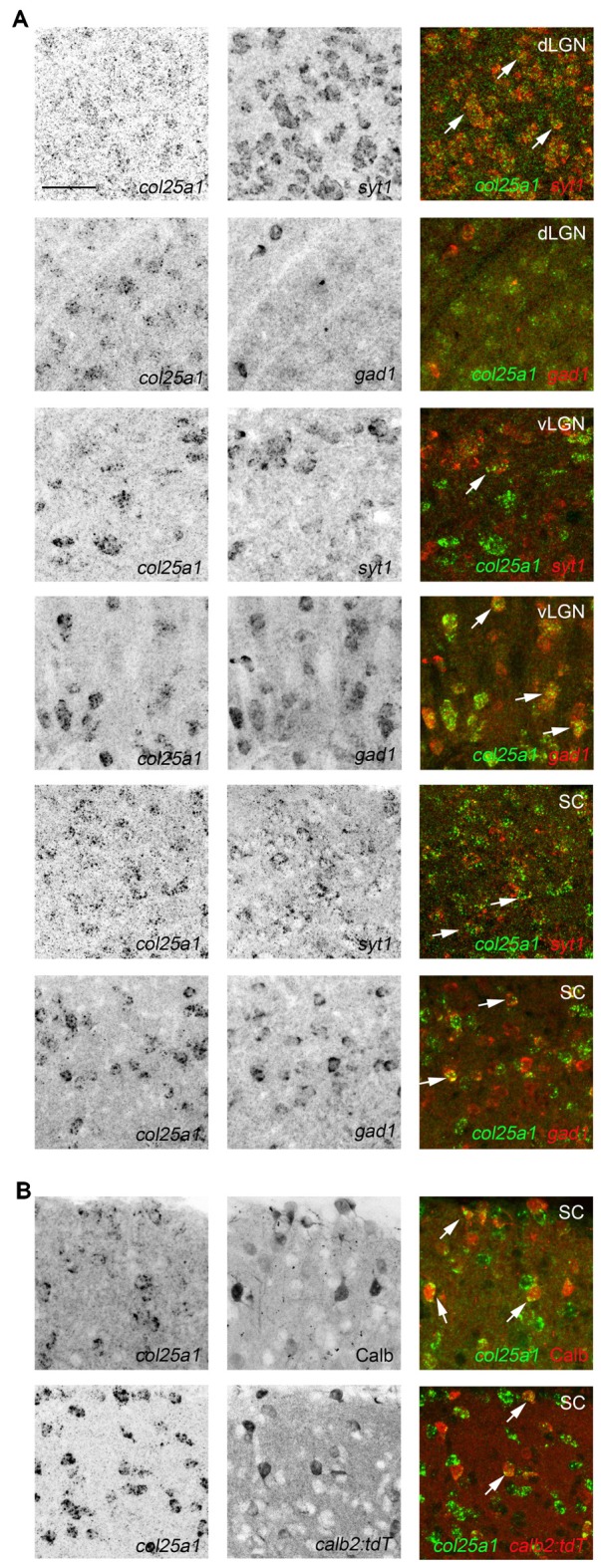
Class-specific expression of *col25a1* by neurons in retino-recipient nuclei. **(A)** Double ISH with riboprobes against *col25a1* and either *syt1* or *gad1* in the dorsal lateral geniculate nucleus (dLGN), vental lateral geniculate nucleus (vLGN) and superior colliculus (SC). Co-expressing cells are depicted with arrows. In dLGN, *col25a1* mRNA is generated in *syt1*-expressing excitatory relay cells but is largely absent from *gad1*-expressing local interneurons. In contrast, in vLGN, *col25a1* expression is largely restricted to *gad1*-expressing inhibitory neurons. In SC, *col25a1* mRNA is generated by both *syt1*- and *gad1*-expressing neurons. **(B)** Localization of *col25a1* mRNA in calbindin (Calb)- and calretinin (Calr)-expressing interneurons in SC. Calb-expressing cells were labeled by immunohistochemistry. Calr-expressing cells were genetically labeled in *calb2-cre:rosa-stop-tdt* reporter mice. Scale bar = 30 μm for **(A,B)**.

To assess whether *col25a1* mRNA was generated by neurons in these retino-recipient regions, a similar D-ISH approach was used as described above for *col23a1*; riboprobes against *col25a1* and either *syt1* or *gad1* were employed. At least in dLGN, vLGN, IGL and SC, *col25a1* appeared to be generated by neurons (Figure 6). The composition of neuronal types varies significantly in these brain regions. For example, a significant portion of neurons in vLGN and IGL are GABAergic and inhibitory, whereas the majority of neurons in dLGN are excitatory (Monavarfeshani et al., 2017). Based on the dense accumulation of *col25a1*-expressing neurons in both regions it seemed likely from the onset of these experiments that unique classes of neurons generated *col25a1* in these retino-recipient regions. Indeed, D-ISH confirmed this to be the case. In dLGN, excitatory *syt1*-expressing relay cells, but not *gad1*-expressing inhibitory neurons, generated *col25a1* (Figure 6). In the adjacent vLGN (and IGL), the opposite appeared true since *col25a1* expression was restricted to *gad1*-expressing cells (Figure 6). Finally, in superior colliculus, subsets of both *syt1* and *gad1-*expressing cells generate *col25a1* mRNA (Figure 6). Moreover, since the superior colliculus contains multiple types of local interneurons we tested whether multiple classes were capable of generating *col25a1* in this region. Indeed, immunostaining for calbindin-expressing interneurons and transgenic labeling of calretinin-expressing interneurons (in *calb2-cre:rosa-stop-tdt* mice) revealed subsets of both interneuron populations generate this MACIT (Figure 6).

**Figure 7:**
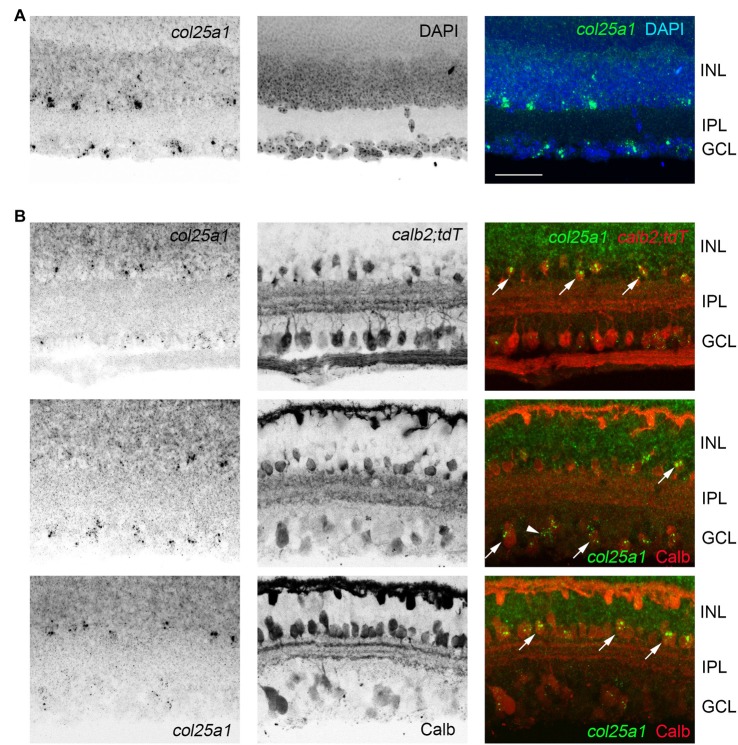
Expression of *col25a1* by amacrine cells in the retina. **(A)** Cellular localization of *col25a1* mRNA by ISH in P21 mouse retina. Nuclei are labeled by DAPI. GCL, ganglion cell layer; INL, inner nuclear layer; IPL, inner plexiform layer. **(B)** Localization of *col25a1* mRNA in calbindin (Calb)- and calretinin (Calr)-expressing amacrine cells and RGCs. Calb-expressing cells were labeled by immunohistochemistry (IHC). Calr-expressing cells were genetically labeled in *calb2-cre:rosa-stop-tdt* reporter mice. Arrow indicate co-expression. Scale bar = 40 μm.

In addition to expression in retino-recipient regions of the mouse brain, sparse expression was observed in cerebral cortex (Figure 5), and, as previously reported (Kay et al., 2011), in the retina (Figure 7). Expression in the retina appeared limited to sparse cells in the inner-most inner nuclear layer (where amacrine cells reside) and in the ganglion cell layer (which contains both retinal ganglion cells and displaced amacrine cells; Figure 7). In both the INL and GCL, *col25a1* expression appeared restricted to subsets of calretinin- and calbindin-expressing retinal neurons (Figure 7).

### *Col25a1* mRNA Is Generated by Interneurons in the Hippocampus

**Figure 8:**
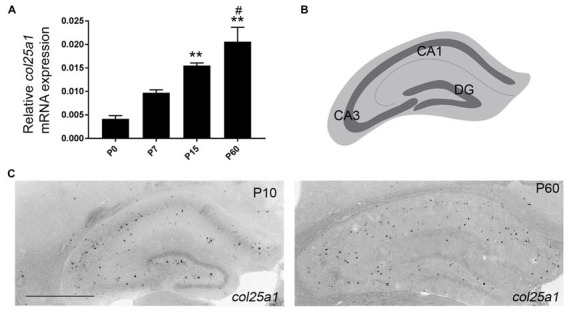
Developmental expression of *col25a1* in mouse hippocampus. **(A)** Quantitative RT-PCR (qPCR) shows a developmental increase in col25a1 expression in mouse hippocampus from hippocampal RNA isolated at P0, P7, P15 and P60. Levels of *col25a1* are normalized to *gapdh*. Data are means +/− SEM. **Indicates expression levels differ from levels at P0 at *P* < 0.001. ^#^Indicates expression level differs from levels at P7 at *P* < 0.01. *n* = 3 mice per age. **(B)** Schematic representation of mouse hippocampus. Dark gray line represents GCL in the dentate gyrus (DG) and stratum pyramidalis in Cornu Ammonis regions (CA1 and CA3). **(C)** Cellular localization of *col25a1* mRNA by ISH in P10 and P60 hippocampi. Scale bar = 500 μm.

Since qPCR and ISH both demonstrated a higher level of *col25a1* mRNA expression in the hippocampus compared with other telencephalic brain regions (Figures 1, 4, 5), we next sought to address the developmental and cell-specific expression of *col25a1* in this region. qPCR analysis showed that expression of *col25a1* appeared relatively low at birth but increased significantly into adulthood (Figure 8A). ISH confirmed this developmental pattern, even though the cell-specific expression of *col25a1* remained relatively sparse and spread-out in the adult hippocampus (Figure 8A).

**Figure 9:**
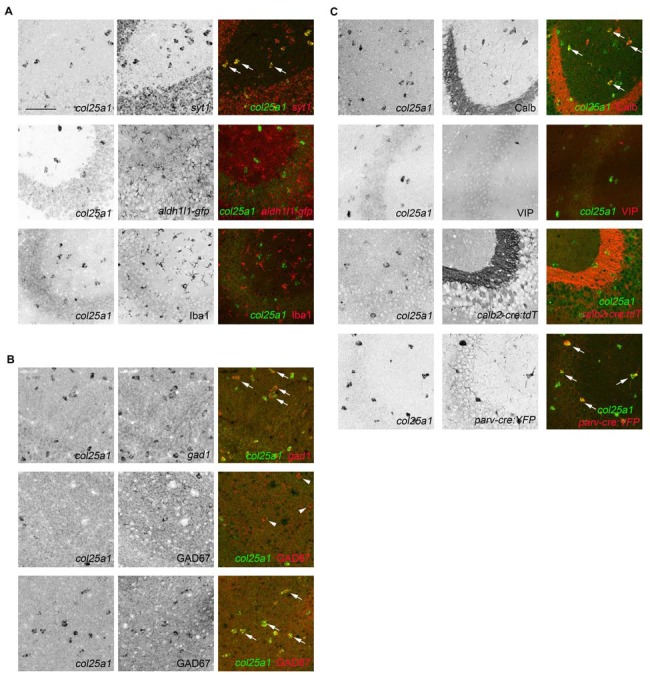
Subsets of hippocampal interneurons express *col25a1* mRNA. **(A)** Hippocampal neurons, but not glia, generate *col25a1* mRNA. *Col25a1* expression is assessed by ISH. Neurons are identified by expression of *syt1* mRNA. Astrocytes are labeled transgenically in *aldh1l1-gfp* mice. Microglia are labeled by Iba1 IHC. Arrows highlight cells co-expressing *col25a1* and *syt1*. **(B)** Interneurons generate *col25a1* mRNA. *Col25a1* expression is assessed by ISH. Interneurons are labeled by the expression of *gad1* mRNA or GAD67 protein. Arrows highlight cells co-expressing *col25a1* and *gad1* or GAD67. Arrowheads indicate GAD67-expressing cells that do not express *col25a1*. **(C)** Expression of *col25a1* by distinct classes of interneurons. *Col25a1* detected by ISH. Calb- and vasoactive intestinal peptide (VIP)-expressing interneurons detected by IHC. Calr- and Parv-expressing interneurons are labeled transgenically in *calb2-cre:rosa-stop-tdt* and *parv-cre:thy1-stop-yfp* mice, respectively. Scale bar = 75 μm for all panels.

The hippocampus is a well-studied telencephalic structure associated with the limbic system of the brain that is subdivided into the dentate gyrus (DG), four Cornu Ammonis areas (CA1-4), and the subiculum (Figure 8B). Within each of these regions, excitatory neurons are clustered together in distinct layers—in the DG, excitatory neurons are clustered in the GCL, and in CA1-4 and subiculum, excitatory neurons are clustered in the stratum pyramidalis. The diffuse distribution of *col25a1*-expressing cells, with some of these cells in stratum pyramidalis or GCL but many more outside of these regions (Figure 8C), led us to suspect that this MACIT collagen was generated by local interneurons or glia. We applied a similar strategy to test whether neurons in the hippocampus generated *col25a1* mRNA as described above—we performed D-ISH with riboprobes against *col25a1* and *syt1*. We also coupled *col25a1* ISH with methods to label glial cells, which included using transgenic tissues in which astrocytes express green fluorescence protein (GFP) or immunolabeling microglia with antibodies against Ionized calcium binding adaptor molecule 1 (Iba1). These studies demonstrated that astrocytes and microglial cells do not generate *col25a1*, and that all *col25a1*-expressing cells co-expressed *syt1* (Figure 9A). Thus, like in other brain regions, this MACIT collagen is generated by neurons (and not glia) in the hippocampus.

It is important to note that not all *syt1*-expressing cells generated *col25a1* (Figure 9A), likely because *syt1* mRNA is generated by both excitatory and inhibitory neurons in the mouse hippocampus (Ullrich et al., 1994; Marquèze et al., 1995; Su et al., 2010). To test whether *col25a1* was generated by inhibitory interneurons, we took two approaches: interneurons were labeled with antibodies against GAD67 or with riboprobes against *gad1*. Both approaches revealed inhibitory interneurons generated *col25a1* (Figure 9B). But again, not all *gad1*-expressing or GAD67-immunoreactive interneurons generated *col25a1*. These findings suggest that only a subset of local interneurons make collagen XXV.

Over 20 different classes of hippocampal interneurons exist and can be divided into classes based upon location within the hippocampus, morphology, electrophysiology, connectivity and neurochemical gene expression (Freund and Buzsáki, 1996; McBain and Fisahn, 2001; Klausberger and Somogyi, 2008). Since our ability to localize *col25a1*-expressing cells was limited to ISH, we used the expression of specific neurochemicals to distinguish which classes of these cells may generate this MACIT collagen. Specifically, we tested whether *col25a1* was generated by calbindin (Calb)-, calretinin (Calr; also, called Calb2)-, parvalbumin (Parv)- and VIP-expressing interneurons. Calb- and VIP-expressing interneurons were labeled with antibodies directed against each of these neurochemicals, whereas Parv- and Calr-expressing interneurons were transgenically labeled in *parv-cre;thy1-stop-yfp* and *calb2-cre;rosa-stop-tdt*, respectively. Coupling each of these tools with ISH revealed that Parv- and Calb-expressing hippocampal interneurons generated *col25a1* (Figure 9C). At the same time, we detected few, if any, *col25a1*-expressing cells that co-expressed any of the other interneuron markers assessed (Figure 9C). Therefore, these studies confirm that select classes of hippocampal interneurons generate *col25a1*.

## Discussion

The past two decades have seen a steady increase in the attention paid to the role of ECM molecules in neural development, repair, and function (Grimpe and Silver, 2002; Dityatev and Schachner, 2003; Dityatev, 2010; Kwok et al., 2011), however roles for collagens in the mammalian brain remain relatively under-explored (Fox, 2008; Hubert et al., 2009). Collagens have likely been overlooked for roles in brain development and function because of the general lack of connective tissue structures and fibrillar collagen in the brain. However, the vast majority of collagen and collagen-like genes encode proteins that are not assembled into fibrillar supramolecular assemblies, and instead, generate ECM and transmembrane molecules with distinct bio-activities (Ricard-Blum, 2011). A series of studies have now revealed roles for these unconventional collagens (and collagen-like molecules) in neural circuit formation and function (Fox et al., 2007; Stevens et al., 2007; Latvanlehto et al., 2010; Su et al., 2010, 2012, 2016; Tanaka et al., 2014; Härönen et al., 2017), motivating us to closely examine the cell-specific and developmental patterns of collagen gene expression in the mammalian brain. Here, we focused our attention on MACITs, a family of transmembrane collagens that includes collagen XIII, XVII, XXIII and XXV. Our results demonstrate that: (1) each MACIT shows region-specific expression patterns in the mammalian brain; (2) *col23a1* is generated by excitatory neurons in the AOB and cells in the INL of the retina; (3) *col25a1* is generated by subsets of both excitatory and inhibitory neurons throughout much of the CNS; and (4) *col25a1* expression in the brain is highest in regions innervated by RGCs.

When comparing these studies with previous studies, an intriguing generalization begins to emerge: all MACITs appear to be expressed in mouse retina, and, perhaps more importantly, each appears to be generated by largely distinct subset of retinal cells. Here, we demonstrate that distinct cell populations in the INL generate *col23a1* and *col25a1*. Additionally, we show expression of *col25a1* in the ganglion layer, a result that matches previous studies that show *col25a1* is a marker for a subset of direction-selective retinal ganglion cells (Kay et al., 2011). In addition to expressing *col25a1*, retinal ganglion cells also appear capable of generating *col13a1* mRNA (Sandberg-Lall et al., 2000), although details regarding cell-specific expression of this MACIT in the retina have not been thoroughly characterized. It is important to highlight that over 30 classes of retinal ganglion cells have been described in the rodent retina (Sanes and Masland, 2015; Baden et al., 2016). Since *col25a1* is generated by less that 10% of all RGCs (Kay et al., 2011) it remains unclear whether *col25a1* and *col13a1* are co-expressed in single retinal ganglion cells or whether discrete classes of RGCs generate each. Collagen XVII, which is structurally the least similar MACIT, is also produced in the retina, but by entirely different cells than collagens XIII, XXIII, and XXV: it is generated by photoreceptors and Muller glial cells (Claudepierre et al., 2005). It is also worth noting that in addition to the diverse, cell-specific expression of MACITs in the retina, results here demonstrate an enrichment in *col25a1* expression in regions of the brain that receive input from RGCs.

*So why might MACITs exhibit such distinct, cell-specific patterns of expression in the retina and retino-recipient regions of brain?* Unfortunately, the answer to this remains unresolved at this time. However, it is well established that unconventional collagens, and especially MACITs, contribute to cell-cell and cell-ECM interactions (Ricard-Blum, 2011). For example, collagen XVII is a component of hemidesmisomes, where it plays critical roles in anchoring cells to the basal lamina by binding laminins (Claudepierre et al., 2005; Powell et al., 2005; Nishie et al., 2011). The shed ecto-domain of collagen XVII is also capable of binding a number of transmembrane adhesion receptors, such as heterodimeric integrins (Nykvist et al., 2001; Löffek et al., 2014; Moilanen et al., 2017), many of which are expressed in the retina (Brem et al., 1994; Leu et al., 2004; Brooks et al., 2013). Similarly, collagen XIII is a component of adherens junctions, focal adhesions, and synaptic adhesions (Peltonen et al., 1999; Hägg et al., 2001; Latvanlehto et al., 2010; Härönen et al., 2017) and it binds both ECM molecules and integrin receptors (Nykvist et al., 2000; Tu et al., 2002; Dennis et al., 2010). Fewer studies have probed collagen XXV interacting partners or receptors. However, it is clear that collagen XXV binds both heparin and amyloid β (Aβ) peptides, which are cleaved fragments of amyloid precursor protein (APP; Osada et al., 2005). Decreased levels of collagen XXV in patients with CCDD has been shown to lead to decreased levels of APP (Shinwari et al., 2015). Interestingly, APP is not only expressed in the mammalian retina, but it is highly enriched in RGCs and has been suggested to be important for the targeting of retinal axons to retino-recipient nuclei within the brain (Osterfield et al., 2008; Nikolaev et al., 2009; Osterhout et al., 2015). These findings, together with emerging evidence that unconventional collagens contribute to circuit formation in the brain (Su et al., 2010, 2012, 2016), raise the interesting possibility that MACITs are another family of adhesion molecules that contribute to the development of the retina and subcortical visual circuits (Sanes and Yamagata, 2009; Zipursky and Sanes, 2010).

Of course, similar roles for these transmembrane collagens may exist in other brain regions, such as the hippocampus and AOB, regions with high levels of cell-specific MACIT expression. But they may just as well play a number of other roles in brain. Unconventional collagens contribute to myelin formation in the peripheral nervous system and gliomedin, a transmembrane collagen-like molecule, mediates axon-Schwann cell interactions and is required for node of Ranvier formation (Eshed et al., 2005, 2007; Maertens et al., 2007; Feinberg et al., 2010). Neuronal expression of MACITs and glial expression of MACIT binding integrins (Colognato and Tzvetanova, 2011; Tanigami et al., 2012) may well contribute to neuro-glial signaling. Brain-derived MACITs may also contribute to the organization of supramolecular assemblies of brain ECM. The most prominent ECM assembly in the brain is the perineuronal net (PNN), a lattice-like matrix of lecticans (a family of chondroitin sulfate proteoglycans), tenascins, hyaluranonin and proteoglycan binding link proteins (HAPLNs) and hyaluran (Zimmermann and Dours-Zimmermann, 2008). PNNs ensheath the somas and proximal processes of select classes of interneurons (Celio and Blümcke, 1994; Celio et al., 1998). Interestingly, PNN-coated neurons are largely the same classes of cells that express collagen XXV in the telencephalon and the developmental upregulation of *col25a1* mRNA in mouse hippocampus coincides with the emergence of PNNs (Levy et al., 2015).

There are clearly more possible roles for transmembrane collagens in the mammalian brain and the studies presented here do little to elucidate such roles. However, the identification that MACITs are generated in both region- and cell-specific manners is a crucial first step in understanding how this unique family of transmembrane collagens may contribute to nervous system development and function.

## Author Contributions

AM, CNK, US, JS and MAF contributed to the design of the experiments. AM, CNK and JS performed ISH and analyzed the associated data. AM and US performed qPCR and analyzed the associated data. AM, JS, CNK and MAF wrote and revised the manuscript. All authors approved the final version of the manuscript.

## Conflict of Interest Statement

The authors declare that the research was conducted in the absence of any commercial or financial relationships that could be construed as a potential conflict of interest.

## Footnotes

^1^http://mouse.brain-map.org
